# The Above-Ground Part of Submerged Macrophytes Plays an Important Role in Ammonium Utilization

**DOI:** 10.3389/fpls.2022.865578

**Published:** 2022-06-06

**Authors:** Ling Xian, Wyckliffe Ayoma Ochieng, Samuel Wamburu Muthui, Duncan Ochieng Otieno, Siwei Yu, Wei Li, Xue Yan, Quan Yu, Fan Liu

**Affiliations:** ^1^Core Botanical Gardens/Wuhan Botanical Garden, Chinese Academy of Sciences, Wuhan, China; ^2^University of the Chinese Academy of Sciences, Beijing, China; ^3^Sino-Africa Joint Research Centre, Chinese Academy of Sciences, Wuhan, China

**Keywords:** ammonium uptake, ammonium translocation, ammonium assimilation, above-ground part, phytoremediation, submerged macrophytes

## Abstract

As a paradoxical nutrient in water ecosystems, ammonium can promote plants growth under moderate concentration, but excess of it causes phytotoxic effects. Previous research has revealed that glutamate dehydrogenase in the above-ground part of submerged macrophytes plays an important role in ammonium detoxification. However, the strategies of ammonium utilization at the whole plant level of submerged macrophytes are still unclear and the role of the above-ground part in nutrient utilization has not been clearly elucidated in previous studies, hence, directly influencing the application of previous theory to practice. In the present research, we combined the methods of isotopic labeling and enzyme estimation to investigate strategies of ammonium utilization by the submerged macrophytes. The results showed that when [NH_4_^+^-N] was 50 mg L^–1^, ^15^N taken up through the above-ground parts was 13.24 and 17.52 mg g^–1^ DW, while that of the below-ground parts was 4.24 and 8.54 mg g^–1^ DW in *Potamogeton lucen*s and *Myriophyllum spicatum*, respectively. The ratios of ^15^N acropetal translocation to uptake were 25.75 and 35.69%, while those of basipetal translocation to uptake were 1.93 and 4.09% in *P. lucen*s and *M. spicatum*, respectively. Our results indicated that the above-ground part was not only the main part for ammonium uptake, but also the major pool of exogenous ammonium. Besides, the dose–response curve of GDH (increased by 20.9 and 50.2% under 15 and 50 mg L^–1^ [NH_4_^+^-N], respectively) exhibited by the above-ground parts of *M. spicatum* indicates that it is the main site for ammonium assimilation of the tolerant species. This study identifies the ammonium utilization strategy of submerged macrophytes and reveals the important role of the above-ground part in nutrient utilization providing new insight into the researches of nutrient utilization by plants and theoretical supports for water restoration by phytoremediation.

## Introduction

Nitrogen is an indispensable nutrient in human life and production. As one of the most important determinants for plant growth, nitrogen fertilizer is widely used to increase crop production. However, only half of the applied nitrogen, on average, is taken up by crops (Kanter et al., 2019). Overapplication of nitrogen fertilizer in agriculture together with the nitrogenous discharge from industrial wastewater and livestock causes a number of environmental pollutions such as the destruction of aquatic ecosystems and biodiversity loss (Galloway et al., 2008; Kanter et al., 2019).

Excess nitrogen in the aquatic ecosystem mainly consists of nitrate–nitrogen and ammonium–nitrogen which is the main inorganic nitrogen available for plants (Lopes and Araus, 2006). As the preferred inorganic nitrogen for plants, ammonium can promote growth under the micromolar concentration range (Gazzarrini et al., 1999), but when the concentration reaches millimolar levels, toxicity will occur (Britto and Kronzucker, 2002; Hachiya and Sakakibara, 2017). Previous research has revealed that ammonium metabolism *via* the pathway catalyzed by GDH (glutamate dehydrogenase) plays an important role in the above-ground part of the submerged macrophytes to ammonium detoxification (Xian et al., 2020). However, whether the above-ground part is the main site for ammonium utilization in submerged macrophytes at the whole plant level is puzzling which directly influences the feasibility of the aforementioned research results in water restoration by phytoremediation.

General conceptions imply that the below-ground tissue of plants is the most important part of nature because most essential nutrients for life such as mineral elements enter the biosphere and food chains through the roots of higher plants (Lamont, 1982). Except for carbon, hydrogen, and oxygen, about 14 indispensable nutrient elements required by plants are mainly taken up through their roots from the soil and transported to the shoots for their growth and development (Jukes, 1995; Che et al., 2018). Specific to submerged macrophytes, multitudinous nutrients exist in both the water column and sediments. They uptake nutrients not only from the sediments but also from the overlying water through their leaves (Stapel et al., 1996; Richter and Gross, 2013; Lin et al., 2017). Besides, the below-ground tissues of the most submerged macrophytes are degenerated; typically, the ratio of below-ground to above-ground tissue of submerged macrophytes is no more than 10% (Kautsky, 1987; Huang et al., 2017). Hence, is the ammonium utilization strategy of submerged macrophytes similar to the general conception? Does the above-ground part of submerged macrophytes matter in ammonium utilization?

Submerged macrophytes are important primary producers in freshwater ecosystems (Moss, 1995; Jeppesen et al., 1998; Fritz et al., 2017). They occupy habitats characterized by a phase boundary of water and sediment which contain different nutrient components and are hard to be simulated. This is due to the lack of a suitable method to separate the nutrient of the above- and below-ground parts for the whole plant level of the submerged macrophytes. The existence of this bottleneck makes it difficult for relevant studies to be conducted. Hence, it is still not clear whether it is either the above-ground part or below-ground part that matters the most in nutrient utilization for submerged macrophytes.

In this study, we designed a test to isolate the nutrient solution surrounding the above-ground from that surrounding the below-ground parts of whole plants of submerged macrophytes. To better understand the strategy of ammonium utilization at the whole plant level of submerged macrophytes, a controlled experiment with different ammonium concentrations was set up using ^15^NH_4_^+^ as the sole nitrogen source for the above- and below-ground parts separately. Our research aimed to reveal the strategy of ammonium utilization at the whole plant level of submerged macrophytes and provide new insight on nutrient utilization in plants, therefore, providing theoretical support and reasonable guidance for the phytoremediation technologies.

## Materials and Methods

### Plant Material and Experimental Site

*Myriophyllum spicatum* L. and *Potamogeton lucens* L. were chosen as experimental materials because of their different sensitivities to ammonium as illustrated in our previous research (Xian et al., 2020). *M. spicatum* is tolerant to high [NH_4_^+^-N] while *P. lucens* is intolerant. Plants were collected randomly from the same colony in Erhai Lake (25°36′–25°58′N, 100°06′–100°18′E), the second-largest freshwater lake with a subtropical plateau monsoon climate, located on the Yungui Plateau, southwest China (Zhu et al., 2018; Zhong et al., 2019). Approximately 10 cm apices of plants without leaves were used for transplantation. After 4 months of cultivation, healthy plants were collected for the laboratory experiment.

### Isolation of Nutrients in Above- and Below-Ground Parts of the Plants

Hermetic bags made with natural emulsion from 15 brands were chosen as the initial material because they are well-sealed and are homogeneous in production. Nutrient leakage from hermetic bags was estimated using high [NH_4_^+^-N] (1,000 mg L^–1^) and deionized water to create hyper-osmosis between the inside and outside. The brand of hermetic bags with the least leakage was chosen as the most suitable material to separate the solution between above- and below-ground parts of the two species (results are shown in Supplementary Figure 1).

### Ammonium-Dosing Experiment in Laboratory

The whole plants of *M. spicatum* and *P. lucens* were collected from cultivation tanks after 4 months of culture and transported in a culture medium at the end of pre-culture using the equipment we designed (Supplementary Figure 2). Our experimental design took account of the fact that submerged macrophytes absorb nutrients mainly from the liquid phase, and the liquid medium can not only provide enough nutrition to the plants, but also ensure the uniformity of nutrition within the culture time. Thus, the culture medium was prepared using 10% Hoagland’s solution with either (^14^NH_4_)_2_SO_4_ or (^15^NH_4_)_2_SO_4_ (δ 98%, Sigma, United States) as the sole nitrogen source. We set two groups: (A) ammonium in solutions for the above-ground parts was prepared by (^15^NH_4_)_2_SO_4_ while that of the below-ground parts was prepared by (^14^NH_4_)_2_SO_4_, (B) ammonium in solutions for the above-ground parts were prepared by (^14^NH_4_)_2_SO_4_, while the below-ground part was prepared by (^15^NH_4_)_2_SO_4_ (Supplementary Figure 3). Ammonium nitrogen concentrations were set according to our previous study (Apudo et al., 2016; Xian et al., 2020), in brief, 3 concentrations (0.1 mg L^–1^, 15 mg L^–1,^ and 50 mg L^–1^) were set for the above-ground part while [NH_4_^+^-N] for the below-ground part was set according to the ratio of [NH_4_^+^-N] in pore water to overlying water from our field investigation of 22 water bodies (Supplementary Table 1). Culture conditions were set at 25°C, with a photoperiod of 14/10-h (light/dark) and light intensity of 108 μmol photons/m^2^/s. The experiment lasted for 4 days in accordance with our previous study (Xian et al., 2020) after which the samples were collected for further analysis.

### Estimation of Parameters

We collected appropriate samples from above- and below-ground parts separately at the end of culture to analyze nitrogen uptake and translocation ability within the two species. After drying by Lyophilizer (Freezone 4.5L, Labconco, United States), samples were sent to Environmental Stable Isotope Laboratory (ESIL), Institute of Environment and Sustainable Development of Agriculture, Chinese Academy of Agricultural Sciences for isotope nitrogen detection. Considering that the water content of submerged macrophyte leaves is much higher than that of terrestrial plants, rapid loss of moisture during the measurement of leaf area would cause significant changes in leaf area. Furthermore, the leaf types of *M. spicatum* and *P. lucens* are quite different which may cause larger errors in the evaluation of the surface area, thus, the content of ^15^N absorption was expressed as ^15^N increment per gram of dry weight (Bouma et al., 2002; Guo et al., 2017; Cao et al., 2018; Svennerstam and Jämtgård, 2022). The ^15^N content in plant tissue was calculated according to the methods of Guo et al. (2017) with minor modifications by the following formulas:


(1) $$\mathrm{Ratio}\,\mathrm{value}\,\left(\delta \right)=\frac{{\text{Ratio}}_{s\,a\,m\,p\,l\,e}-{\text{Ratio}}_{s\,t\,a\,n\,d\,a\,r\,d}}{{\text{Ratio}}_{s\,t\,a\,n\,d\,a\,r\,d}}\times 1000$$

(2) N15⁢(mg⁢g-1)=Ratios⁢a⁢m⁢p⁢l⁢e1+Ratios⁢a⁢m⁢p⁢l⁢e×N⁢content⁢in⁢samplesample⁢dry⁢weight

The natural ^15^N (0.365%) of atmospheric N_2_ was used as the Ratio*_*standard*_*.

To determine the plant state, we measured the concentration of dissolved oxygen (DO), chlorophyll fluorescence, and total chlorophyll content under experimental conditions according to the methods used in our previous studies (Apudo et al., 2016; Xian et al., 2020). To better understand the strategy of nitrogen allocation and ammonium assimilation between the above- and below-ground parts, we quantified the contents of soluble proteins, free amino acids (FAAs), and the activities of enzymes associated with ammonium assimilation according to Xian et al. (2020).

### Statistical Analysis

Statistical analysis was carried out with SPSS software version 22.0. Student’s *t*-test was performed to compare the differences in photosynthetic parameters, ^15^N uptake and translocation, nitrogen nutrient and activities of enzymes between the two species under the same ammonium concentration. One-way ANOVA was applied to analyze the effects of ammonium concentrations on all the parameters. Tukey test was used for *post hoc* comparisons for all the analyses. Significance level for all tests was set at *p*-value < 0.05. Data shown in figures are presented as mean ± SD from five independent replicates for each concentration.

## Results

### Differences in Ammonium-Nitrogen Uptake and Translocation

**FIGURE 1:**
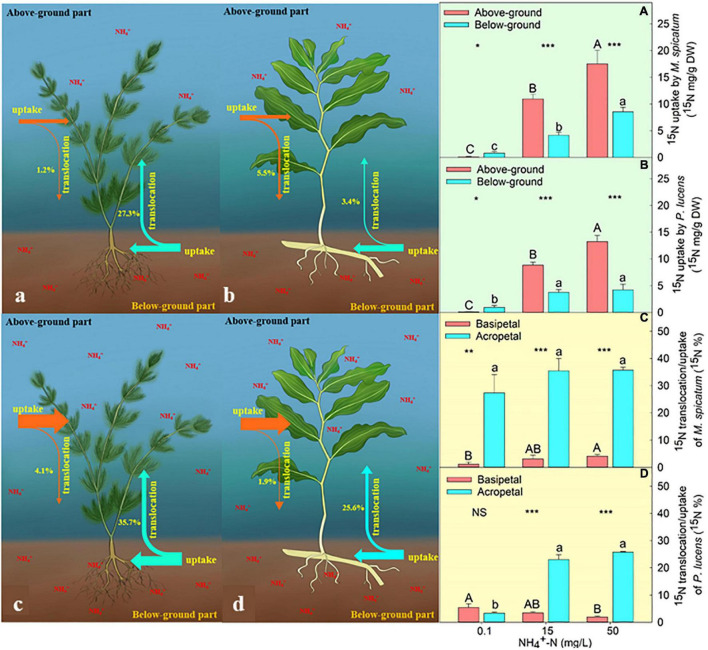
Characteristics of ^15^N uptake and translocation in plants. Schematic diagram of ^15^N uptake and translocation of *M. spicatum* and *P. lucens* under normal **(a,b)** and high **(c,d)** [NH_4_^+^−N]. The effects of different [NH_4_^+^−N] on the ^15^N uptake of *M. spicatum* **(A)** and *P. lucens* **(B)** through the above-ground part (red bar) and the below-ground part (blue bar) and ^15^N translocation of basipetal (red bar) and acropetal (blue bar) of *M. spicatum* **(C)** and *P. lucens* **(D)**. Different letters represent significant differences (*p* < 0.05). Statistical Student’s *t*-tests are shown, *p < 0.05, **p < 0.01, ***p < 0.001, NS: no significance.

We found that both the above- and below-ground parts of *M. spicatum* and *P. lucens* have the ability to take up external ammonium (Figures 1A,B). Under normal [NH_4_^+^-N] (0.1 mg L^–1^), both species exhibited the highest uptake ability of below-ground part (Figures 1A,B). However, with the increase in [NH_4_^+^-N], nitrogen uptake ability of above-ground part was always higher than that of the below-ground part in both the species (Figures 1A,B). The content of ^15^N uptake through the above-ground part was 1.4- and 2.1-folds higher than the below-ground parts for *P. lucens* under 15 and 50 mg L^–1^ [NH_4_^+^-N], respectively, while that of *M. spicatum* was 1.6- and 1.1-folds higher for the same concentrations.

We further analyzed the translocation of nitrogen from exogenous ammonium–nitrogen between above- and below-ground parts by comparing the proportion of ^15^N translocation to uptake content. During the 4-day culture, both species exhibited two directions of nitrogen translocation (from above to below and *vice versa*) (Figures 1C,D). Under normal [NH_4_^+^-N] condition (0.1 mg L^–1^), the acropetal and basipetal translocation of ^15^N in *P. lucens* did not show any significant differences (Figure 1D). However, the acropetal translocation was significantly higher than basipetal in *M. spicatum* (Figure 1C). Under high [NH_4_^+^-N] (≥15 mg L^–1^), acropetal translocation dominated in both species (Figures 1C,D). At 15 mg L^–1^ [NH_4_^+^-N], ^15^N acropetal translocation/uptake was about 23.0 and 35.5% while basipetal was about 3.5 and 3.1% in *P. lucens* and *M. spicatum*, respectively. When ammonium was excess (50 mg L^–1^), ^15^N acropetal translocation/uptake was about 25.8 and 35.7% while basipetal movement was about 1.9 and 4.1% in *P. lucens* and *M. spicatum*, respectively.

### Estimation of Physiological Parameters

**FIGURE 2:**
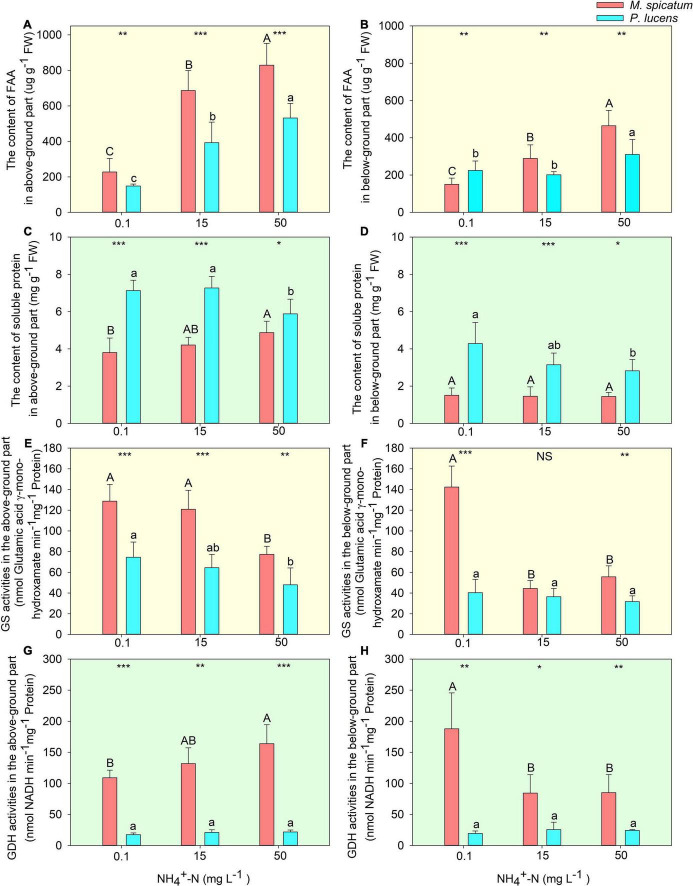
Estimation of nitrogen allocation and ammonium assimilation in plants. The effect of [NH_4_^+^-N] on the content of FAA **(A,B)**, soluble protein **(C,D)**, enzymes activities of GS **(E,F)**, and GDH **(G,H)** in the above- and below-ground parts of *M. spicatum* (red bar) and *P. lucens* (blue bar). Different letters represent significant differences (*p* < 0.05), capital letters: *M. spicatum*, lowercase letters: *P. lucens*. Statistical differences between the two species are designed as follows: *p < 0.05, **p < 0.01, ***p < 0.001, NS stands for no significance, based on the Student’s *t*-tests.

With an increase in [NH_4_^+^-N], no significant differences were witnessed in the photosynthesis of *M. spicatum*, however, a considerable decrease was exhibited by *P. lucens* (Supplementary Figure 4). A significant increase in FAA could be observed in both above- and below-ground parts of the two species with an increase in [NH_4_^+^-N] (Figures 2A,B). Soluble proteins in the two species displayed different tendencies. For *M. spicatum*, it increased with the increase in [NH_4_^+^-N] concentration in the above-ground part but no significant changes were noted in its below-ground part. However, a significant decrease was observed in both the above- and below-ground parts of *P. lucens* (Figures 2C,D). Through enzymatic estimation, we found that with an increase in ammonium concentration, a decline in GS activities was detected in the above-ground parts of the two species (Figure 2E). However, different changes were observed in GDH activities of the above-ground parts of the two species: a sharp rise in *M. spicatum* and stable activities in *P. lucens* (Figure 2G). Compared with 0.1 mg L^–1^ [NH_4_^+^-N], the activity of GDH in the above-ground of *M. spicatum* was increased by 20.9 and 50.2% under 15 and 50 mg L^–1^ [NH_4_^+^-N] treatments, respectively. For below-ground parts, a significant decline was exhibited in both GS and GDH of *M. spicatum*, however, no significant changes were witnessed in *P. lucens* (Figures 2F,H).

## Discussion

Nutrient utilization by plants has always been a major hotspot in ecological researches, furthermore, the importance of the below-ground part has been emphasized for a long time. However, whether submerged macrophytes in freshwater ecosystems still follow the general strategy (in which the below-ground part dominates in nutrient utilization) is still unknown. In this research, new findings were revealed where the above-ground parts of submerged macrophytes have been found to play a much bigger role in nutrient utilization than was previously thought.

### Uptake and Translocation of Nitrogen From Exogenous Ammonium

The use of isotopic labeling in this study demonstrated that both species uptake ammonium mainly through their below-ground parts under normal [NH_4_^+^-N] (0.1 mg L^–1^). This strategy is similar to the one found in terrestrial plants where nutrient uptake is mainly through the below-ground part. Due to the limitation of nutrients in the overlying water column for above-ground parts, submerged macrophytes may solely take up nutrients from sediments where the concentrations are sufficient to meet plant’s requirements. However, based on our study, the above-ground parts of submerged macrophytes occupy a great position in ammonium uptake, especially under conditions of high [NH_4_^+^-N] (15 mg L^–1^). Although abundant pieces of evidence have shown that the above-ground parts (e.g., leaves, stems, fruits) have the ability to take up nutrients (Bouma et al., 2002; Fernandez and Brown, 2013), studies comparing uptake capacities between the below-ground and above-ground parts are rare, especially in freshwater ecosystems. The above-ground part has always been the key center of research for submerged macrophytes; it is not only the main site of photosynthesis but also the part in contact with the water environment. In this study, the same strategy of ammonium uptake was exhibited by both *M. spicatum* and *P. lucens*, which reveals the importance of above-ground parts in nutrient uptake (because uptake through the below-ground part only dominates when the nutrient of above-ground part is limited). This may be due to the fact that the contact surface between the above-ground part and water is much larger than the below-ground part and the flowing water also increases the collision between nutrients and plant tissues, which makes it easier for the above-ground part to obtain nutrients from the environment. Furthermore, nutrients taken up through the above-ground part are directly added into the plant’s metabolism, which could potentially and readily influence plant’s growth than nutrients from sediments (Sparks, 2009). This process cannot only supply nutrients to the below-ground part when nutrients in sediment are limited but also improves plants’ tolerance when suffering from the excess nutrient supply (Yang et al., 2009; Guha et al., 2010; Khan et al., 2010). The below-ground part of submerged macrophytes is capable of taking up nutrients although its main function is plant’s anchorage and energy storage to guarantee plant growth. Since the below-ground parts of most submerged macrophytes are degenerated, they are often characterized by relatively smaller root systems compared to their terrestrial counterparts (Kautsky, 1987; Huang et al., 2017). For this reason, many species of submerged macrophyte have been found to survive very well even without the below-ground parts, for instance, *Ceratophyllum demersum*.

Plants transfer nutrients to different organs through a series of processes to meet their growth requirements. In this study, when [NH_4_^+^-N] is 0.1 mg L^–1^, different strategies of nitrogen translocation are observed in *P. lucens* and *M. spicatum*, thereby revealing different nitrogen requirements for the above- and below-ground parts of the two species. Under normal conditions, plants that uptake and store nutrients for their growth and reproduce asexually are considered to be more advantageous in mild habitats (Williams, 1975; Grant, 1981; Li, 2014). For *P. lucens*, its rhizome (the below-ground part) is the most common vegetative propagule, thus, the storage of nutrients in the below-ground part provides enough energy to support its reproduction. However, the shoot fragment is the main vegetative propagule for *M. spicatum*. Therefore, more nutrients are required for the above-ground part which makes the acropetal translocation dominant in *M. spicatum*. Nevertheless, under high [NH_4_^+^-N] (≥ 15 mg L^–1^), the acropetal translocation dominates in the allocation of nitrogen, which makes the above-ground part to be the main nitrogen pool. For terrestrial plants, transpiration in the leaves is the main driving force for mineral element translocation (Campbell et al., 1999; Tanner and Beevers, 2001), thus, the main translocate direction of most mineral nutrients is acropetal. It is interesting that the translocation pattern for the submerged macrophytes under high-ammonium concentrations is similar to that in the terrestrial plants. However, the main driving force for the submerged macrophytes may result from water pressure, nutrient concentrations, and also other reasons which are beyond the scope of this study. Except for the driving force, there are two main reasons that could be used to explain this result. First, sexual reproduction is considered to be more advantageous in heterogeneous or challengeable environments (Williams, 1975; Grant, 1981). Plants have to maintain their population through sexual reproduction under harsh environments (Li, 2014), which forces the above-ground part to store more nutrients and energy required for blossom formation, pollination, seed and fruit development, etc. Second, the above-ground part of plants is not only the main site for photosynthesis but also the main area for energy synthesis. In addition, it provides the most suitable place for plants to perform and maintain their physiological functions efficiently (Piao et al., 2020). When sufficient nutrients are present in water, submerged macrophytes uptake considerably enough amounts through their above-ground parts and assimilate them directly. This could reduce energy consumption in nutrient translocation from the below-ground to the above-ground parts and improve efficiency of energy utilization.

### Ammonium Assimilation and Nitrogen Allocation

Although the strategies of ammonium uptake and translocation in *M. spicatum* and *P. lucens* are much similar under conditions of high-ammonium concentrations, different adaptations to the changeable environment can be found between the two species: high tolerance in *M. spicatum* and sensitivity in *P. lucens*. This could be explained by the strategy of ammonium assimilation and nitrogen allocation in plant tissue.

Ammonium absorbed by plants is assimilated through glutamine synthetase (GS) and glutamate dehydrogenase (GDH) (Glevarec et al., 2004; Skopelitis et al., 2006; Fontaine et al., 2012; Xian et al., 2020), afterward, transamination and synthesis produce new amino acids and protein. In this research, the significant increases in FAA and soluble protein in the above-ground tissue of *M. spicatum* reveal its strong ability to assimilate ammonium, which is supported by an increase in GDH activity in the above-ground part. GDH is a stress-responsive enzyme (Zhou et al., 2015) and catalyzes the reversible reaction that converts ammonium to glutamate; the biosynthesis direction of GDH plays an important role when ammonium is in excess (Abiko et al., 2010; Xian et al., 2020). The other enzyme, GS, displayed a sharp decline in both above- and below-ground parts of *M. spicatum* under excess [NH_4_^+^-N] (50 mg L^–1^). This may be the strategy of submerged plants to save energy because GS catalyzes a more energy-consuming pathway for ammonium assimilation; it costs about 18% more energy than the pathway catalyzed by GDH (Helling, 1994, 1998; Geisseler et al., 2009). Furthermore, GS is an enzyme with a high affinity to ammonium, the enzyme plays a dominant role in ammonium assimilation when ammonium is in limited supply, however, the activity is inhibited when ammonium is in excess (Zhou et al., 2017). The same tendency of GS activity can be observed in the above-ground part of *P. lucens*, whereas, the activity of GDH in both the above- and below-ground part did not show many differences under various ammonium concentrations. This may be due to the limitation of energy [e.g., Fv/Fm, total chlorophyll content and DO in solutions decreased under high [NH_4_^+^-N] (≥ 15mg L^–1^)] and nitrogen accumulation which makes the ability of ammonium assimilation to decrease, causing toxicity of ammonium in *P. lucens* especially under excess [NH_4_^+^-N] (50 mg L^–1^), followed by the degradation of protein and the increase of FAA exhibited in both the above- and below-ground tissues (Skopelitis et al., 2006; Xian et al., 2020).

### The Investigation of Ammonium Detoxification Mechanism in *Myriophyllum spicatum*

In our research, *M. spicatum* exhibited higher plasticity in its physiology under various ammonium concentrations in comparison with *P. lucens*. The higher capacity of photosynthesis efficiently transforms light energy into chemical energy which improves plants’ ability to balance C:N ratios in their tissue, thus improving the tolerance of plants subjected to excessive ammonium environments (Xiao et al., 2007; Apudo et al., 2016). Furthermore, the major site (above-ground part) of ammonium uptake and assimilation helps plants to avoid energy wastage in nutrient translocation. Moreover, the energy-efficient pathway catalyzed by GDH, not only plays an important role in ammonium detoxification (Xian et al., 2020), but also improves the conversion efficiency of ammonium to organics in *M. spicatum*. Hence, *M. spicatum* possesses a stronger ability than *P. lucens* to adapt to changeable environments which supports it survival in conditions characterized with excess ammonium.

## Conclusion

As one of the most suitable candidates for water restoration in phytoremediation, submerged macrophytes are still enigmatic in many aspects such as nutrient uptake, translocation, and assimilation. In this research, we found that both the above- and below-ground parts of the two species could uptake ammonium from the environment, meanwhile, the acropetal and basipetal translocation of nitrogen were exhibited in both the species. However, ammonium uptake through the above-ground part and the acropetal translocation were dominant in both species when ammonium is in excess. The ammonium-tolerant species, *M. spicatum*, has higher plasticity in photosynthesis and ammonium utilization under various [NH_4_^+^-N] than *P. lucens*, moreover, the pathway catalyzed by GDH in the above-ground part of *M. spicatum* contributes more in ammonium detoxification.

This study explored the characteristics of ammonium utilization in two representative species of submerged macrophytes and firstly found the importance of the above-ground part in ammonium utilization under high-ammonium concentrations. Hence, we propose that more attention should be paid to the above-ground part of submerged macrophytes in phytoremediation for water restoration, and we suggest the use of *M. spicatum* with luxuriant above-ground part as a candidate for phytoremediation in the restoration of water bodies polluted by ammonium. The results not only provide theoretical support for the research on ammonium utilization of submerged macrophytes but also offer technical support for water restoration by the submerged macrophytes.

## Data Availability Statement

The raw data supporting the conclusions of this article will be made available by the authors, without undue reservation.

## Author Contributions

LX performed the experiments and wrote the manuscript. WL designed the research and reviewed the manuscript. LX and FL analyzed the data. LX and SY drew the figures. WO and SM participated in the collection of samples. WO, SM, DO, and XY reviewed the manuscript. FL conceptualized the study and obtained financial support for the work. All authors revised and approved the manuscript.

## Conflict of Interest

The authors declare that the research was conducted in the absence of any commercial or financial relationships that could be construed as a potential conflict of interest.

## Publisher’s Note

All claims expressed in this article are solely those of the authors and do not necessarily represent those of their affiliated organizations, or those of the publisher, the editors and the reviewers. Any product that may be evaluated in this article, or claim that may be made by its manufacturer, is not guaranteed or endorsed by the publisher.
