# Supplementary material for: The Above-Ground Part of Submerged Macrophytes Plays an Important Role in Ammonium Utilization
Source: Front Plant Sci. 2022 Jun 6;13:865578. doi: 10.3389/fpls.2022.865578 (PMC9207443; doi:10.3389/fpls.2022.865578)
Supplement: Supplementary file 1 [file Data_Sheet_1.DOCX]

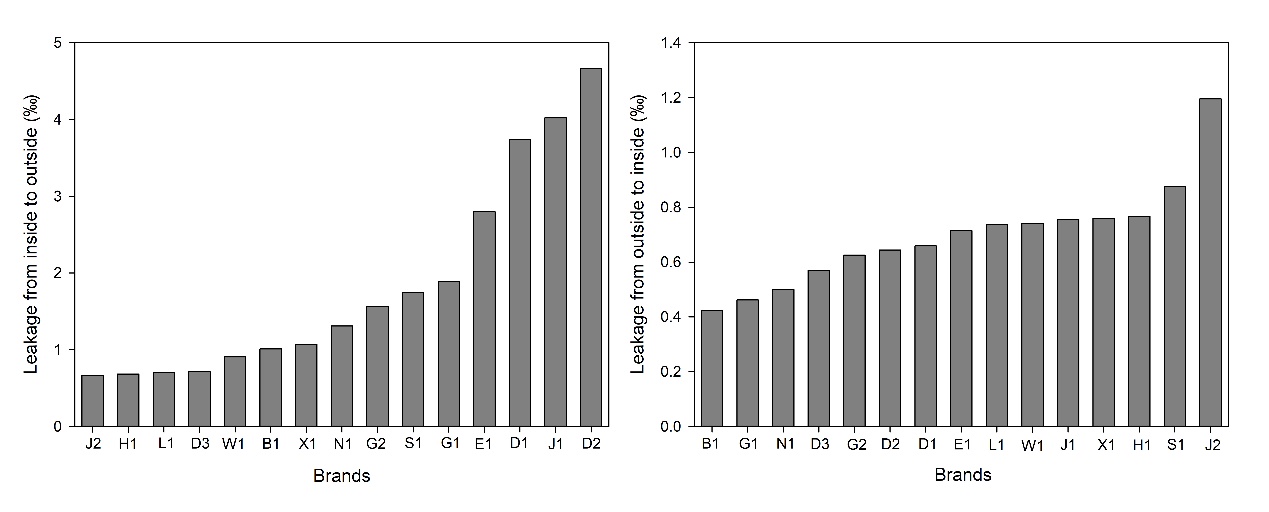


**Figure S1. Evaluation of hermetic bags.** Leakage of ammonium from inside to outside **(A)** and from outside to inside **(B)** of hermetic bags. Brands are shown as abbreviations: B1-SHUANGDIE, D1-DUREX1, D2-DUREX2, D3-DUREX3, E1-DAXIANG, G1-OKAMOTO1, G2-OKAMOTO2, H1-HESETIANXIANG, J1-JISSBON1, J2-JISSBON2, L1-LASTING, N1-NANZIHAN, S1-SIXSEX, W1-WEIWEILEISA, X1-XINBOLE.


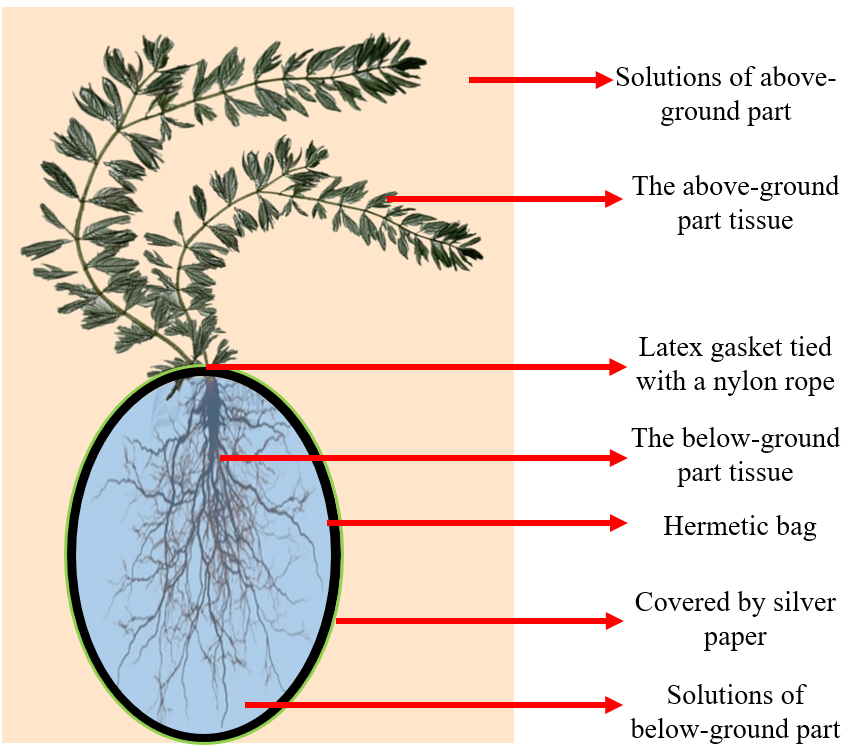


**Figure S2. Schematic diagram of the equipment designed to separate the nutrients of the above from below-ground parts.**


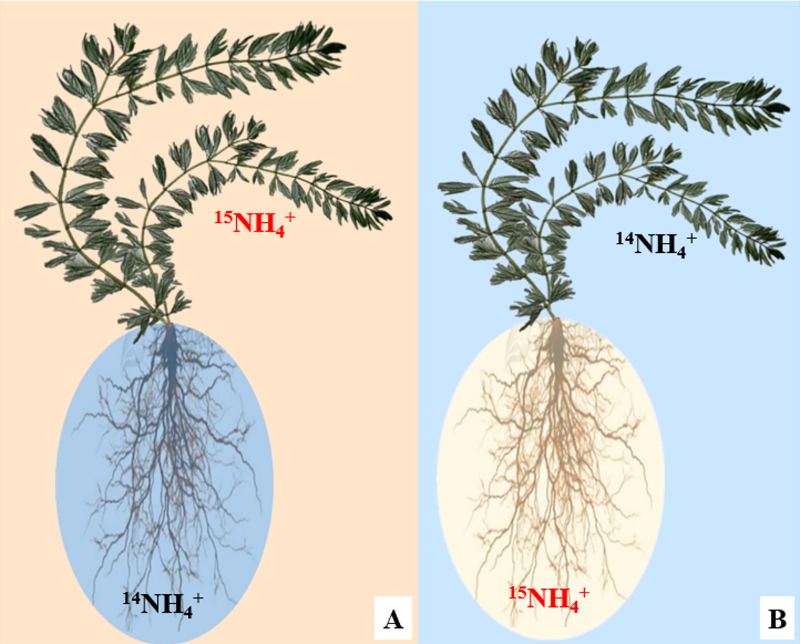


**Figure S3. Schematic diagram of ammonium in solutions.** **(A)**, above-ground part--^15^NH_4_^+^, below-ground part--^14^NH_4_^+^; **(B)**, above-ground part--^14^NH_4_^+^, below-ground part--^15^NH_4_^+^


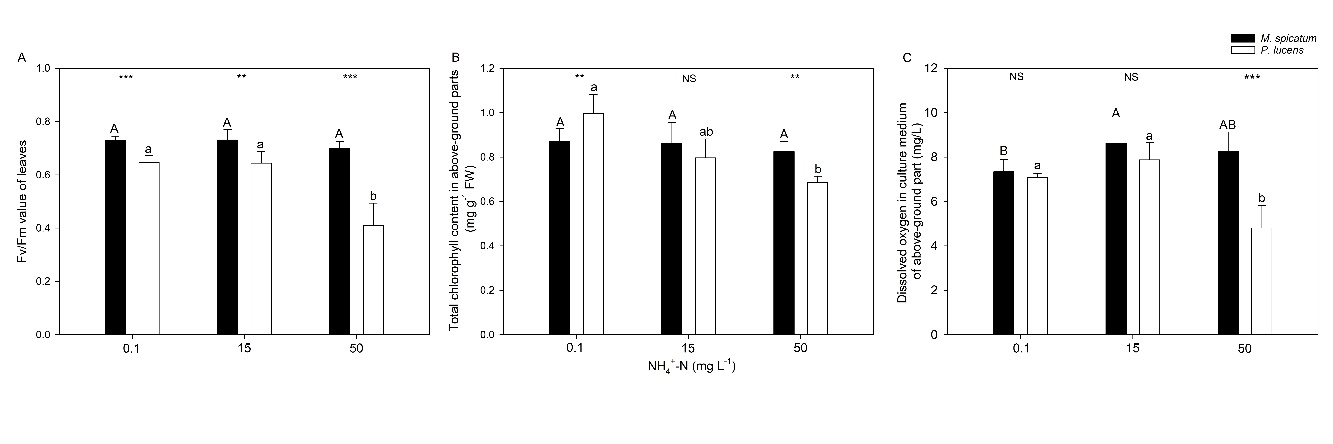
**Figure S4.** **Evaluation of plants’ state under different treatments:** Effect of different [NH_4_^+^-N] on the Fv/Fm values **(A)**, total chlorophyll content **(B)** and concentration of dissolved oxygen in above-ground parts **(C)** of *M. spicatum* (black bar) and *P. lucens* (white bar). Different letters represent significant differences (p < 0.05), capital letters: *M. spicatum*, lowercase letters: *P. lucens*. Statistical Student’s t-tests are shown, *p<0.05, **p<0.01, ***p<0.001, NS no significance.
