# Supplementary material for: The Above-Ground Part of Submerged Macrophytes Plays an Important Role in Ammonium Utilization
Source: Front Plant Sci. 2022 Jun 6;13:865578. doi: 10.3389/fpls.2022.865578 (PMC9207443; doi:10.3389/fpls.2022.865578)
Supplement: Supplementary file 2 [file Table_1.docx]

**Table S1.** Ammonium-nitrogen concentrations of overlying water and pore water in 22 water bodies.

| Name | Overlying water | Pore water | Pore water/Overlying water | ratio average |
| --- | --- | --- | --- | --- |
|  | NH_4_^+^-N (mg L^-1^) | NH_4_^+^-N (mg L^-1^) | [NH_4_^+^-N] ratio |  |
| Manhe River | 0.50 | 18.01 | 35.77 | 24.81 |
| Hejialou River | 0.70 | 7.55 | 10.82 |  |
| Honghu Lake | 0.30 | 7.20 | 24.02 |  |
| Shitanghu Lake | 0.34 | 6.81 | 19.93 |  |
| Shimenhu Lake | 0.08 | 2.98 | 39.23 |  |
| Quantanxia River | 0.01 | 0.23 | 31.00 |  |
| Dahu Lake | 0.52 | 20.26 | 38.60 |  |
| Lianhu Lake | 0.96 | 9.47 | 9.86 |  |
| Linghu Lake | 0.21 | 20.96 | 99.01 |  |
| Xixiaohu Lake | 2.82 | 29.15 | 10.34 |  |
| Zhongziwan River | 0.23 | 3.44 | 14.62 |  |
| Xingyunhu Lake | 0.38 | 3.74 | 9.95 |  |
| Fuxianhu Lake | 0.20 | 5.67 | 29.02 |  |
| Changqiaohai Lake | 0.22 | 11.80 | 53.79 |  |
| Datunhai Lake | 3.77 | 26.12 | 6.93 |  |
| Erhai Lake | 0.08 | 2.53 | 33.03 |  |
| Daqinghe River | 0.96 | 10.10 | 10.54 |  |
| Baoxianghe River | 2.77 | 96.11 | 34.67 |  |
| Yandonghu Lake | 0.71 | 9.91 | 14.00 |  |
| Zhuzihu Lake | 0.19 | 1.64 | 8.63 |  |
| Jiutianxuannv Lake | 0.66 | 3.75 | 6.26 |  |
| Niushanhu Lake | 0.33 | 1.94 | 5.88 |  |
